# Sir E. T. Cook's "Florence Nightingale"

**Published:** 1913-11-15

**Authors:** 


### SIR E. T. COOK'S "FLORENCE NIGHTINGALE."

### The Real Character of the Lady with the Lamp.*

This two-volume biography will come as a healthy and a richly deserved shock to most people, inside and outside the institutional world, for it proves authoritatively and once for all that the Lady with the Lamp legend was chiefly the invention of Longfellow, and certainly the least characteristic and unique trait of Florence Nightingale. The greatest merit of Sir Edward Cook's "Life" is that this much-misunderstood legend is seen at last in its proper perspective.

Blue-stocking, globe-trotter, society girl, reader of Latin and Greek, tourist, haunter of Owenite "advanced" book-shops, "new woman," rebel in the home—all these phrases are far more characteristic of Florence Nightingale's mind and heart, for her heart was in her mental life, than the somewhat sickly, anæmic figure which, under the pious cloak that Longfellow's sentimental imagination spun for him, has formed the popular heroine; so different from the real person who Queen Victoria said possessed such a clear head that "I wish we had her at the War Office."

Indeed, so much do the facts alter the grains of truth out of which the legend sprang, that we have thought it worth while to devote a preliminary notice wholly to the astringent facts of the case, leaving the hospital and nursing reforms which Miss Nightingale initiated to subsequent and separate consideration. True as the above facts are—indeed if they were not true the great literature of Europe during the latter half of the nineteenth century would not have portrayed the types in which it delighted—the actual part which the heart, and the respect for the emotions, did play in Florence Nightingale's spiritual economy is of the highest interest. If we turn to the Crimean episodes in her history we find that the epithet of "ministering angel" was simply the flower of grateful hearts which had felt the strength and value of a strong administrator, of an experienced hand, of a trained organiser. As, however, the men, patients and officials alike, could not reconcile the possession of such able qualities as these with the womanly ideal then current, they blessed her for the practical hard work which she performed, as none else could, and rewarded her by seizing on a quality which satisfied their imaginations better than the real facts were able to do. It reminds one of the late Mr. W. T. Stead's attitude to Marie Bashkirtseff. With this undoubted psychological basis, the "ministering angel" becomes a fellow-creature (instead of a lay figure to hang sermons on), and is shown to advantage, even from the merely warm-hearted point of view, in her attitude on what became known as "The Battle of the Nurses."

Without going over the old story again, old, indeed, to us, since The Hospital's part in the fray is recalled in the volumes before us, the fact remains that Florence Nightingale opposed nurse registration because she feared that a close corporation would get the nursing profession into its hands and enforce a standard which left "the ministering angel" qualities out. The Hospital then, as now, was on the side of the angels, and nothing is finer than the recognition, by a woman of Miss Nightingale's high mental powers, of the fact that no examination and no training or certificate can make any woman the sort of person whom one cares to have in a sick room when one is ill. That was the sort for which she worked and which her addresses and example inculcated. There must be, she said, "a vocation" for nursing, and without vocation no training would avail. This truth is as needed as ever, and supplies still the final argument against the value of registration to nurses, while its reverse is the insistence of proper training in recognised institutions. Already more hospitals grant certificates than provide training, and only because it is the easier thing to do. With registration the vocational, the spirit of nursing, might be extinguished as a practical ideal for good and all.

She herself wrote that three careers were open to her: the literary life, the married life, and the hospital life. The reason why the first was abandoned lay in the characteristic mystic trait which regards literature as merely the handmaid of life, and not a life at all, apart from its usefulness. Of course, no line can be drawn, but Florence would rarely have written at all if she did appreciate the function of Blue-books. Why she never married the daily papers have abundantly explained. The man who presented himself had every quality except the conscious inner wish to co-operate in her aims. She therefore became the apostle of unmarried women. In the hospital life she invented a new profession for her sex.

In conclusion, one last fact emerges which is worth attention to students of Florence Nightingale's character; the combination in her of the imaginative dreamer (she wrote a novel!) and the practical administrator. Only one recognised psychological type produces this combination. That is the mystic. St. Catharine of Siena, diplomatist, statesman, and visionary; St. Theresa ("undaunted daughter of desires," with her "large draughts of intellectual day"), writer of a handbook for novices ("The Way of Perfection"), foundress of institutions and successful Orders, and society woman enough to use her great social charms to prevent herself from being burnt for heresy. This combination is the highest mystic type. That it flourished in England during the nineteenth century in Florence Nightingale, student of Plato, is amazing and comforting enough. The converse side of the picture is that symbol, for so she has become, Nora Helmer in Ibsen's "Doll's House."

* "The Life of Florence Nightingale," by Sir Edward Cook. In two volumes. Price 30s.

